# Predicting the Effectiveness of Hepatitis C Virus Neutralizing Antibodies by Bioinformatic Analysis of Conserved Epitope Residues Using Public Sequence Data

**DOI:** 10.3389/fimmu.2018.01470

**Published:** 2018-06-27

**Authors:** Vanessa M. Cowton, Joshua B. Singer, Robert J. Gifford, Arvind H. Patel

**Affiliations:** MRC-University of Glasgow Centre for Virus Research, Garscube Campus, Glasgow, Scotland, United Kingdom

**Keywords:** neutralizing antibodies, hepatitis C virus, vaccine, bioinformatics, HCV-GLUE, sequence conservation

## Abstract

Hepatitis C virus (HCV) is a global health issue. Although direct-acting antivirals are available to target HCV, there is currently no vaccine. The diversity of the virus is a major obstacle to HCV vaccine development. One approach toward a vaccine is to utilize a strategy to elicit broadly neutralizing antibodies (bNAbs) that target highly-conserved epitopes. The conserved epitopes of bNAbs have been mapped almost exclusively to the E2 glycoprotein. In this study, we have used HCV-GLUE, a bioinformatics resource for HCV sequence data, to investigate the major epitopes targeted by well-characterized bNAbs. Here, we analyze the level of conservation of each epitope by genotype and subtype and consider the most promising bNAbs identified to date for further study as potential vaccine leads. For the most conserved epitopes, we also identify the most prevalent sequence variants in the circulating HCV population. We examine the distribution of E2 sequence data from across the globe and highlight regions with no coverage. Genotype 1 is the most prevalent genotype worldwide, but in many regions, it is not the dominant genotype. We find that the sequence conservation data is very encouraging; several bNAbs have a high level of conservation across all genotypes suggesting that it may be unnecessary to tailor vaccines according to the geographical distribution of genotypes.

## Introduction

Hepatitis C virus (HCV), a member of the *Flaviviridae* family, is a major cause of liver disease worldwide. Recent estimates indicate that HCV infects approximately 71 million people globally (1). Approximately 70% of infected individuals develop a chronic infection that can lead to liver cirrhosis and hepatocellular carcinoma (HCC). Termed a “silent killer,” the initial infection is usually asymptomatic and individuals are often unaware that they carry the infection until symptoms develop several decades later. In recent years, a number of effective direct-acting antiviral (DAA) drugs have been developed. However, the silent nature of initial infection makes timely diagnosis and treatment more challenging. The long period of chronic infection may already have caused irreversible liver damage or initiated a chain of events that will ultimately result in HCC even if the virus is successfully cleared by DAA-treatment post-diagnosis (2, 3). Further studies are required to address this question. This and other factors including cost, access to treatment, and reinfection enforces the pressing need for a prophylactic vaccine for HCV.

One of the major barriers to vaccine development for HCV is the sequence diversity of the virus. Currently, there are seven genotypes and 67 subtypes that have at least 33 or 15% nucleotide variation, respectively (4). As a result, an effective vaccine must be capable of protecting against challenge by an extremely diverse viral population. The question is how to design such a vaccine? HCV has two surface glycoproteins E1 and E2 that form a heterodimer. These proteins govern the entry process of the virus. The E2 glycoprotein, which contains the receptor-binding site (RBS) for the cellular receptors CD81 and SR-BI is the most studied (5, 6). E2 contains a number of variable regions; hypervariable region 1 (HVR1) is located at the N-terminus (aa384-427), this region has been shown to be important for interaction with the SR-BI receptor and to play a role in antibody evasion by shielding epitopes and preventing neutralization (7–12). The roles of the other variable regions are less defined, they are hypervariable region 2 (aa461-481) and the intergenotypic variable region (aa570-580) (8, 13). E2 has ~11 N-linked glycosylation sites that form a glycan shield, which has also been shown to be involved in immune evasion (14). An insight into possible targets for HCV vaccine development, i.e., surface-exposed, conserved regions of the HCV glycoproteins can be gleaned from studies into broadly neutralizing antibodies (bNAbs). Viral neutralizing antibodies have been shown to inhibit infection by either blocking interaction with the RBS or by inhibition of the post-entry fusion mechanism (15, 16). By definition, bNAbs do this by targeting highly conserved regions within the viral glycoproteins that are involved in these processes. We generated the HCV bNAb, AP33 in 2001 and demonstrated in 2005, with the development of the HCV pseudoparticle (HCVpp) system that it was able to neutralize particles decorated with diverse HCV E1E2 glycoproteins (17, 18). Since then, there has been significant progress in the isolation and characterization of HCV bNAbs, as reviewed by Ball et al. (19). The majority of HCV bNAbs have been shown to target the E2 glycoprotein particularly the CD81 RBS. Within the literature, several different nomenclatures are used to describe these regions, herein, we will use Epitopes 1–4 (20, 21). The potential of utilizing HCV bNAbs to inform rational vaccine design and the associated challenges has been the topic of recent reviews (22, 23). With the plethora of HCV bNAbs now available, which of these would be the most promising for further analysis and vaccine design? In this study, we have probed a large HCV sequence dataset to determine the level of conservation of each bNAb epitope. Using this data and documented neutralization studies, we conclude that the most promising candidates to date as a starting point for development of a bNAb-based vaccine approach are HC84.20, AR4A, 1:7, A8 and AP33. 95-2, HCV1, and Hu5B3.v3 also have strong potential, but there are insufficient neutralization data available at this time.

## Materials and Methods

### Epitope Identification

For each bNAb, the epitope reported in the literature cited was used. For the majority of bNAbs, this was straightforward; however, for a small group, the data in different publications were conflicting. We have used an eight-residue binding motif for bNAb AR3C that differs from the original epitope identified by alanine-scanning (24). These eight residues were consistent between two subsequent reports; the crystal structure of AR3C bound to core E2 and also in an extensive alanine-scanning study (25, 26). AR3A, AR3B, and AR3D were excluded from our analysis as the alanine-scanning data were conflicting and no structural data were available to corroborate either study. We have also updated the binding motifs of several conformational bNAbs (HC84.20, HC84.24, HC84.26, HC-1, HC-11) reported by the Foung lab to incorporate a later study by Pierce and coworkers that includes a comprehensive E1E2 alanine-scanning study (27). Residues that inhibited binding by at least 80% were selected as critical-binding residues. Crucially, as these antibodies bind conformational epitopes, alanine mutation may alter the overall structure of E1E2; therefore, mutations in regions that affected binding of all conformational antibodies were not included.

### Bioinformatic Analysis

The analysis of public HCV sequence data was performed within Genes Linked by Underlying Evolution (GLUE) (28). GLUE is an open source, data-centric bioinformatics environment specialized for the analysis of virus genomic sequence data.

GLUE was used to create a public sequence data resource called HCV-GLUE (28) for the study of HCV genomes. HCV-GLUE provides an interactive web application for public use; the underlying dataset may be also downloaded to a local computer. This dataset currently contains approximately 92,000 HCV sequences derived from the public GenBank database (29) and is updated on a daily basis. Sequences from non-human hosts, <500 bases in length, recombinant, or patent-related are excluded from the set. Within HCV-GLUE, each sequence is assigned a genotype and where possible a subtype according to a maximum likelihood method based on the scheme proposed by Smith et al. (4). Furthermore, each sequence is maintained in alignment to a closely related reference sequence. The GLUE software system provides basic functions for the analysis of amino acid residues across sets of stored sequences. A residue numbering scheme proposed by Kuiken et al. (30) is used within HCV-GLUE.

GLUE allows existing projects such as HCV-GLUE to be extended to address-specific research questions. For the current article, we created an extension, HCV-NABS, which may be downloaded from https://github.com/giffordlabcvr/HCV-NABS. The HCV-NABS extension augments the HCV-GLUE dataset with data relating to 38 neutralizing antibodies and their putative-binding locations. We then also created scripts within the HCV-NABS extension to analyze the frequency of amino acid residue patterns both at individual binding locations and at combinations of binding locations pertaining to each bNAb. Procedures were also added to report the numbers of sequences within each genotype containing a substantial part (90%) of the E2 region of the HCV genome. These data were stratified according to the country of origin, which had been annotated in the GenBank record, if any. We used the HCV-GLUE characterization of sequence genotypes and subtypes to stratify the analysis. The bioinformatics analysis may be reproduced by installing GLUE, HCV-GLUE, and the HCV-NABS extension on any computer.

## Results

### Identification of the bNAb Epitopes

**Figure 1:**
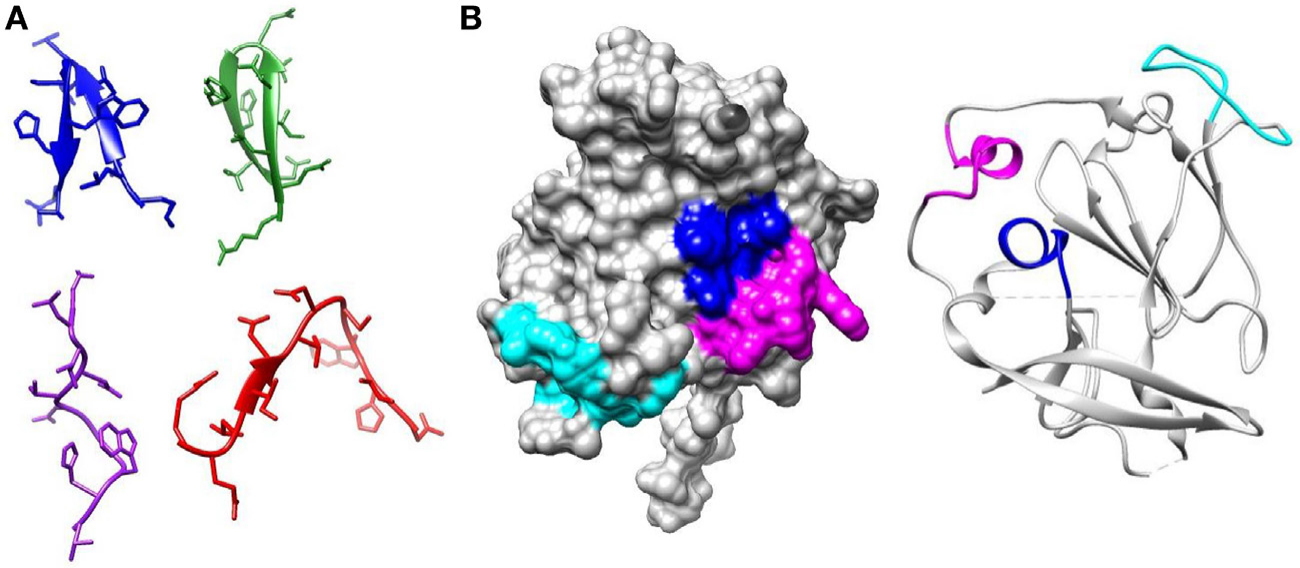
Regions of E2 targeted by broadly neutralizing antibodies. **(A)** Epitope 1 (412–423) is flexible. The structure of this region has been solved bound to several broadly neutralizing antibodies. In AP33 (blue) (PDB 4GAG) and HCV1 (green) (PDB 4DGV), this region forms a β-hairpin structure. In HC33.1 (red) (PDB 4XVJ), it has an intermediate structure between a β-hairpin and a coil and in 3/11 (purple) (PDB 4WHT), it has an extended conformation. **(B)** The core E2 structure (PDB 4MWF) with Epitope 2 (434–446) in magenta, Epitope 3 (525–535) in cyan, and Epitope 4 (611–617) in blue.

**Figure 2:**
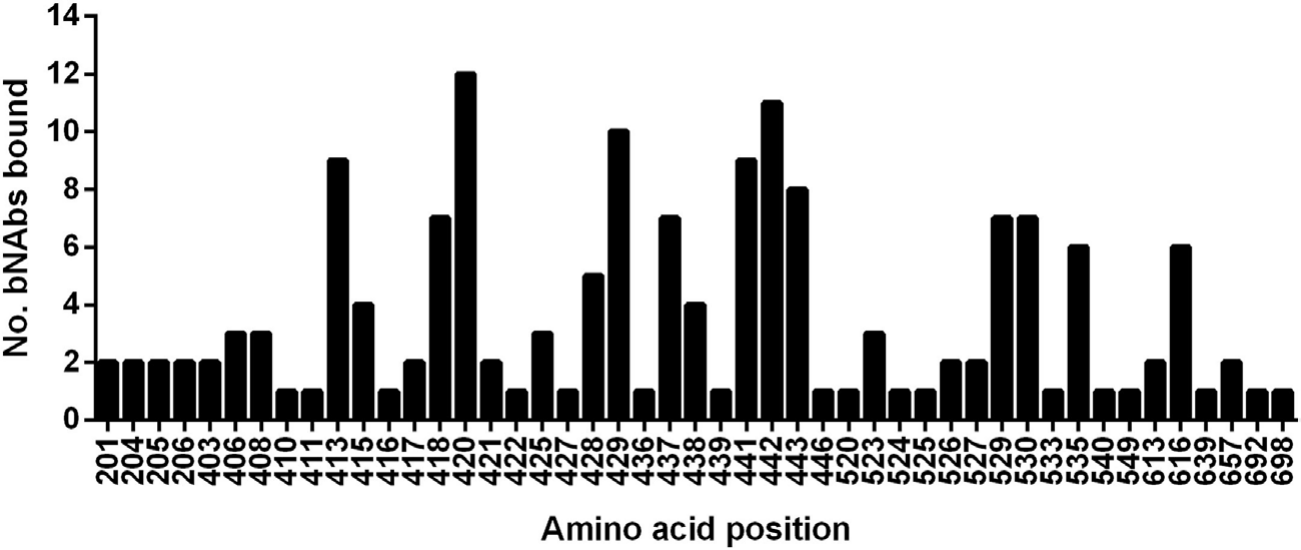
Relative usage of residues bound by broadly neutralizing antibodies. The graph plots the number of broadly neutralizing antibodies in this study that use each amino-acid as part of their epitope.

A group of 38 monoclonal antibodies that has been shown to have broad neutralization activity were selected from the literature. Importantly, the epitopes of this group of antibodies have been characterized by alanine-scanning mutagenesis and/or structural analysis. The bNAbs have been isolated and characterized by many different groups. However, often several antibodies were isolated from the same source as indicated by the nomenclature. The largest such group is the HC84 group; these are all designated as HC84.xx and tend to share overlapping epitopes. The bNAbs used in this study are shown in Table 1, together with the region of E1E2 that they target and the specific residues that are critical for antibody binding. It is well documented that most neutralizing antibodies target particular regions of the E2 glycoprotein that are involved in CD81 binding; Epitope 1 (aa412–423), Epitope 2 (aa434–446), Epitope 3 (aa523–535), and Epitope 4 (aa611–617) as shown in Figure 1 (20, 21). We compared the specific residues bound by all 38 bNAbs and identified 47 E1E2 residues, 27 of which lie within these four epitopes. Certain residues seem to be key target residues as they are recognized by several bNAbs from different sources these include; W420 in Epitope 1 and F442 in Epitope 2 that are targeted by 12 and 11 bNAbs, respectively (Figure 2). We have shown that W420 is a critical residue modulating interactions with the cellular receptors CD81 and SR-BI (31). Other residues only form part of the epitope for 1 bNAb, for instance, T416, A439, and D533.

**Table 1:** Broadly neutralizing antibodies and their epitopes analyzed in the study.

Name	E2-binding residues	Region targeted	Identification of residues	Reference
AR4A	*Y201, T204, N205, D206, R657, L692, D698*	E1E2	Mutagenesis	(32)
AR5A	*Y201, T204, N205, D206, R639, R657*	E1E2	Mutagenesis	(32)
J6.36	*F403, G406*	Hypervariable region 1 (HVR1)	Mutagenesis	(33)
J6.103	*F403, G406*	HVR1	Mutagenesis	(33)
H77.16	*G406, N410, I411*	HVR1	Mutagenesis	(33)
HC33.4	*K408*, L413, W420	HVR1, Epitope 1	Mutagenesis	(34)
HC33.8	*K408*, L413, G418, W420	HVR1, Epitope 1	Mutagenesis	(34)
HC33.29	*K408*, L413, G418, W420	HVR1, Epitope 1	Mutagenesis	(34)
AP33	L413, N415, G418, W420	Epitope 1	Structure	(17, 35, 36)
Hu5B3.v3	L413, N417, W420, I422	Epitope 1	Structure	(37)
HC33.1	L413, G418, W420	Epitope 1	Structure	(34, 38)
HC33.32	L413, G418, W420	Epitope 1	Mutagenesis	(34)
HCV1	L413, N415, G418, W420	Epitope 1	Structure	(39, 40)
95-2	L413, W420	Epitope 1	Mutagenesis	(39)
H77.39	N415, N417	Epitope 1	Mutagenesis	(33)
3/11	N415, W420, H421	Epitope 1	Structure	(41–43)
Mab24	T416, G418, W420, H421	Epitope 1	Mutagenesis	(44)
HC84.22	W420, *N428, C429*, W437, L441, F442, Y443, W616	Epitope 1, 2, and 4	Mutagenesis	(27, 45)
HC84.23	W420, *N428, C429*, W437, L441, F442, Y443, W616	Epitope 1, 2, and 4	Mutagenesis	(27, 45)
AR3C	*T425, N428, C429*, L438, L441, F442, Y443, W529	Epitope 2 and 3	Structure	(24–26)
e20	*T425, L427, N428*, W437, F442, W529, G530, D535, W616	Epitope 2 and 3	Mutagenesis	(46–48)
HC-11	*T425, N428, C429*, G436, W437, L438, F442, Y443, D520, G530, D535	Epitope 2 and 3	Mutagenesis	(27, 49)
HC-1	*C429*, W529, G530, D535	Epitope 3	Mutagenesis	(49, 50)
HC84.20	*C429*, L441, Y613, W616	Epitope 2 and 4	Mutagenesis	(27, 34)
HC84.21	*C429*, L441, F442, Y443	Epitope 2	Mutagenesis	(27, 34)
HC84.24	*C429*, F442, Y443	Epitope 2	Mutagenesis	(27, 34)
HC84.25	*C429*, L441, F442, W616	Epitope 2 and 4	Mutagenesis	(27, 34)
HC84.27	*C429*, L441, F442, Y443, K446, W616	Epitope 2 and 4	Structure	(27, 34, 51)
mAb#8	W437, L438	Epitope 2	Structure	(52, 53)
mAb#41	W437, L438	Epitope 2	Peptide mapping	(52)
CBH-2	W437, A439, G530, D535	Epitope 2 and 3	Mutagenesis	(49)
HC84.1	L441, F442	Epitope 2	Structure	(34, 51)
HC84.26	L441, F442	Epitope 2	Mutagenesis	(34)
1:7	G523, T526, Y527, W529, G530, D535	Epitope 3	Mutagenesis	(54)
A8	G523, T526, Y527, W529, G530, D535	Epitope 3	Mutagenesis	(54)
MAb44	G523, P525, *N540, W549*, Y613	Epitope 3 and 4	Mutagenesis	(44)
J6.27	A524, W529	Epitope 3	Mutagenesis	(33)
H77.31	W529, G530, D533	Epitope 3	Mutagenesis	(33)

*^a^Italicized residues are not in Epitopes 1–4. Numbering is according to the H77 polyprotein. Antibodies are listed in numerical order according to the first residue of their epitope*.

### Analysis of the Level of Conservation of bNAb Target Residues

**Figure 3:**
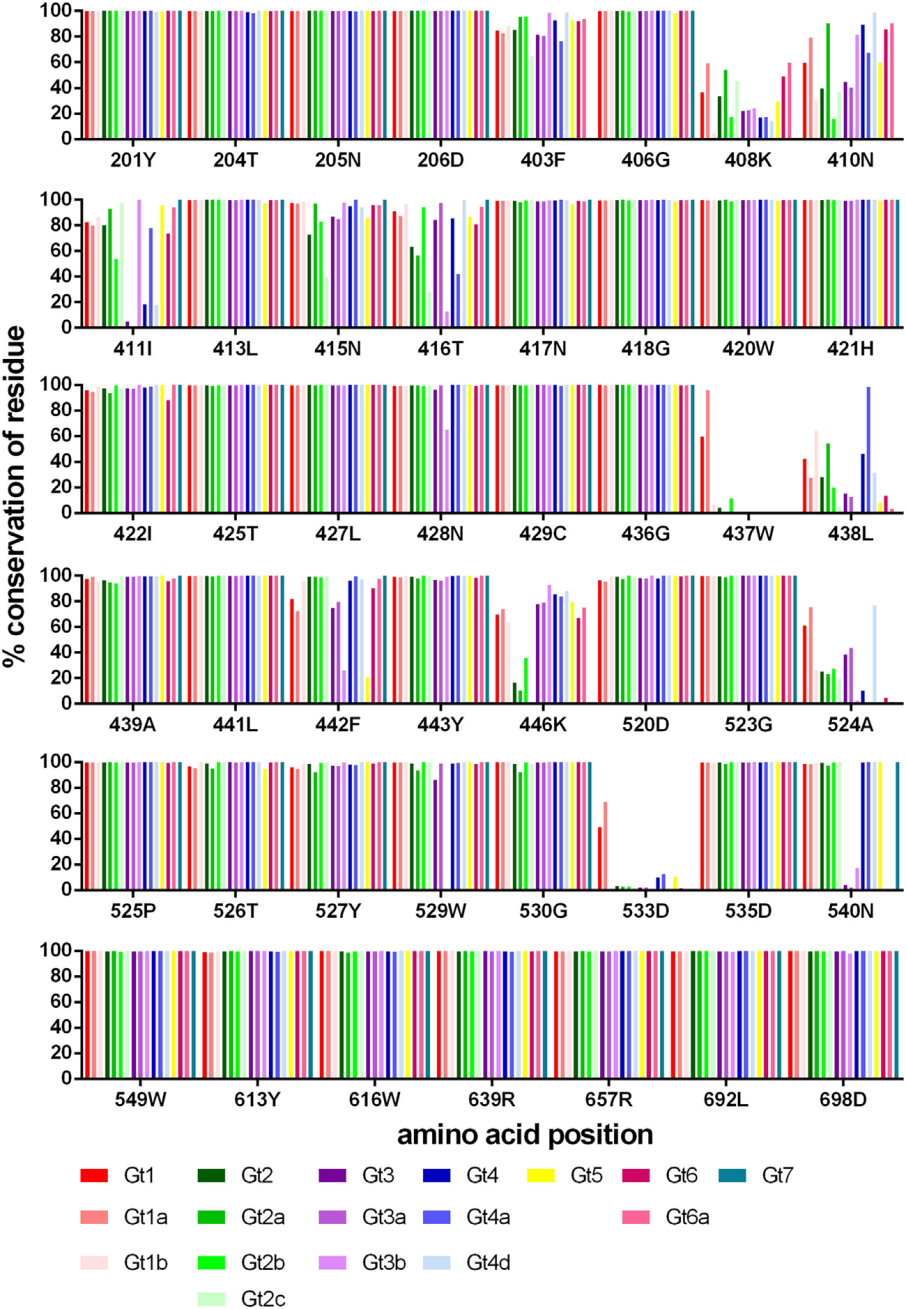
Conservation of bNAb-bound residues. The level of conservation (%) with respect to the reference sequence is shown for each genotype and subtype. Gt1 (red), Gt2 (green), Gt3 (purple), Gt4 (blue), Gt5 (yellow), Gt6 (pink), and Gt7 (teal).

Rather than focusing on the designated epitope regions, we determined the level of conservation of all 47 residues recognized by HCV bNAbs, as a significant number of the antibody-interacting residues are outside these regions (refer to Table 1 for details). We used the HCV-GLUE to analyze the level of conservation for each genotype (1–7) and 10 subtypes (Figure 3; Table S1 in Supplementary Material). For the majority of bNAbs alanine-scanning mutagenesis in the genotype (gt), 1a H77 strain was used to determine the bNAb antibody-interacting residues. The J6 group of bNAbs is the exception as this was mapped using the gt2a J6 virus strain. Consequently, in our analysis, we used the sequence that was used to map the antibody interaction as the reference sequence. Predictably, the overall level of conservation among these residues, which are bound by bNAbs, was high. Positions 408K, 410N in HVR1 and 411I just downstream were less well conserved. In Epitope 1, threonine at position 416 is substituted by serine in a large proportion of gt2a, gt2c, gt3b, and gt4a. In Epitopes 2 and 3, two residues 437W and 533D were well-conserved for gt1 and gt1a but were found to be preferentially replaced by a similar residue, phenylalanine, and glutamic acid, respectively, in all other genotypes and subtypes. Likewise, at position 438, the leucine residue found in the H77 gt1a sequence was an isoleucine in the majority of sequences. Some variants were very genotype specific. In gt2, position 446K was generally serine, arginine, or asparagine depending on the particular subtype. The majority of gt3 and gt6 sequences have a glutamic acid residue replacing the asparagine at position 540; however, in the subtype gt3b, this is commonly a threonine residue. Both variants are particularly interesting as substitution of N540 removes a potential N-linked glycosylation motif at this position.

**Figure 4:**
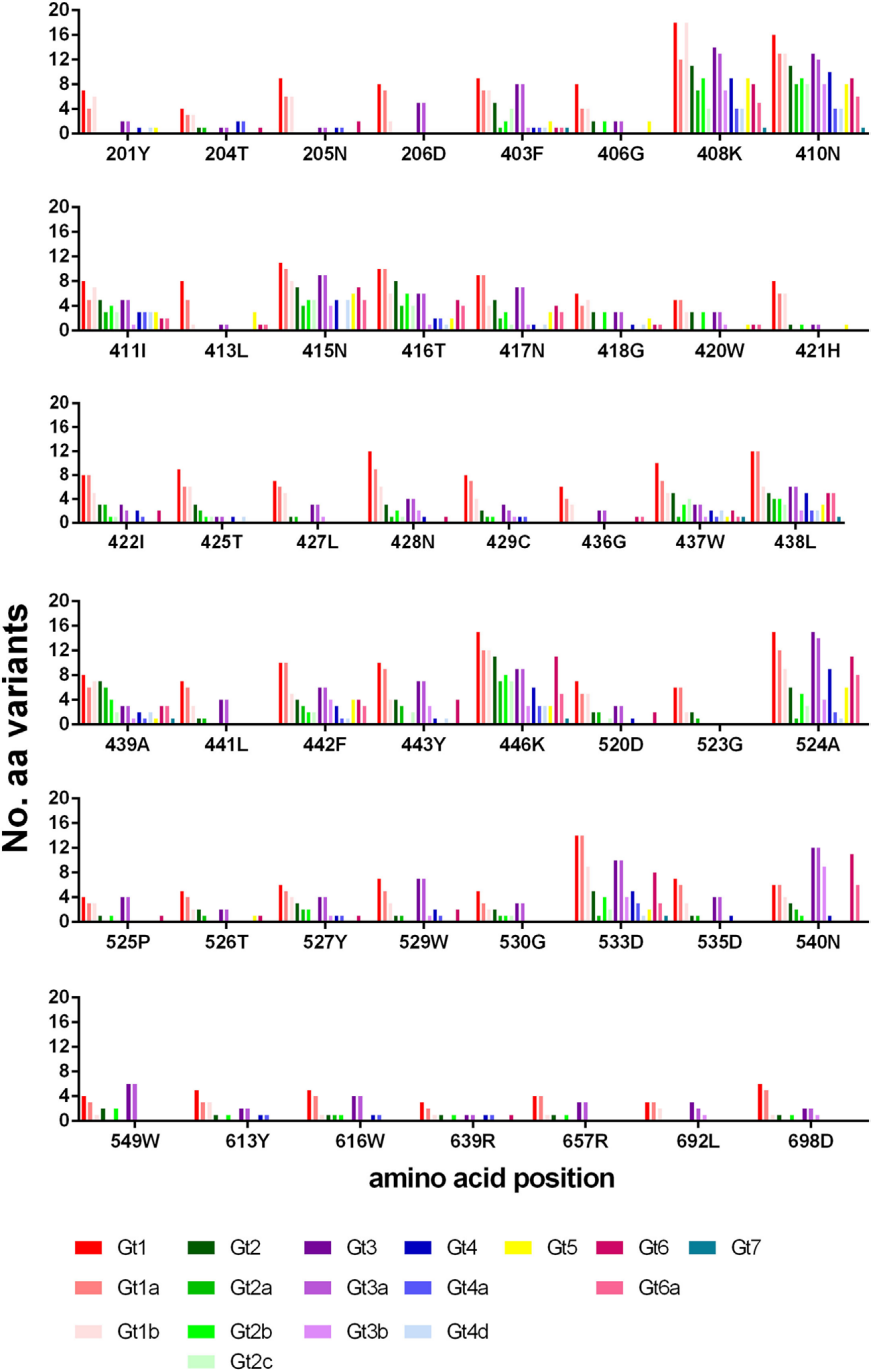
Variance of bNAb bound residues. The number of different amino-acid variants for each residue is shown across the different genotypes and subtypes. Gt1 (red), Gt2 (green), Gt3 (purple), Gt4 (blue), Gt5 (yellow), Gt6 (pink) and Gt7 (teal).

The number of amino-acid variants identified (excluding that of the reference sequence), for each position is shown in Figure 4. Generally, gt1 has a greater range of variants for each position, although this may be skewed due to the large number of gt1 sequences (>23,000) in the database compared to the other genotypes. Even so, it is clear from the data that certain positions are less tolerant of variation than others, most notably residues within E1 (201–206) and toward the E2 C-terminal end (613, 639, 692, 698). Interestingly, of the other six positions whereby nearly all the genotypes/subtypes have fewer than three variants namely 406, 413, 421, 436, 523, 526, three of these are glycine residues.

### Analysis of the Level of Conservation of bNAb Epitopes

To assess how well the complete binding motif of each bNAb was conserved, we determined the residue with the lowest level of conservation relative to the reference sequence for each motif (refer to Table 2). There is the possibility that a particular residue may be differentially conserved between genotypes and therefore, this was done for all seven genotypes. These values were combined to give the % conservation for all genotypes for each binding motif, and this figure was used to rank the bNAb panel from the most conserved overall (HC84.20) to the least conserved (mAb#8, mAb#41 and HC-11). The number of genotypes where the binding motif was conserved by at least 90% is also reported in Table 2. Epitopes of six antibodies had this level of conservation in all seven genotypes. In contrast, the epitope residues of 15 antibodies did not reach this degree of conservation in the glycoprotein of any genotype. The level of conservation varied with respect to each genotype. The epitopes of gt4 E1E2 had the highest level of conservation, with 22/38 antibodies displaying >90% conservation of their binding motifs. This compares to only 7/38 antibodies in the case of gt3, suggesting that this genotype may be the most difficult to neutralize with this bNAb panel.

**Table 2:** The lowest conservation (%) for each bNAb epitope binding motif across genotypes.

Name	Gt1% Con	Gt2% Con	Gt3% Con	Gt4% Con	Gt5% Con	Gt6% Con	Gt7% Con	Sum% Con	No. gt >90%	Region targeted	Rank
AR4A	99.79	99.78	99.72	99.15	99.65	99.86	100	697.95	7	E1E2	2
AR5A	99.79	99.78	99.8	99.15	99.65	99.65	100	697.82	7	E1E2	3
J6.36	85.02	85.28	81.48	92.85	92.76	91.93	0	529.32	3	Hypervariable region 1 (HVR1)	21=
J6.103	85.02	85.28	81.48	92.85	92.76	91.93	0	529.32	3	HVR1	21=
H77.16	59.4	39.75	4.8	18.6	2.72	25.16	0	150.43	0	HVR1	29
HC33.4	36.58	33.51	21.96	16.88	29.53	49.3	0	187.76	0	HVR1, Epitope 1	25=
HC33.8	36.58	33.51	21.96	16.88	29.53	49.3	0	187.76	0	HVR1, Epitope 1	25=
HC33.29	36.58	33.51	21.96	16.88	29.53	49.3	0	187.76	0	HVR1, Epitope 1	25=
AP33	97.57	72.57	86.88	94.94	85.82	95.62	100	633.4	4	Epitope 1	11=
Hu5B3.v3	96.05	97.19	97.24	98.05	96.30	88.03	100	672.86	6	Epitope 1	8
HC33.1	99.66	99.56	99.85	99.87	97.06	99.83	100	695.83	7	Epitope 1	5=
HC33.32	99.66	99.56	99.85	99.87	97.06	99.83	100	695.83	7	Epitope 1	5=
HCV1	97.57	72.57	86.88	94.94	85.82	95.62	100	633.4	4	Epitope 1	11=
95-2	99.73	99.56	99.9	100	97.06	99.83	100	696.08	7	Epitope 1	4
H77.39	97.57	72.57	86.88	94.94	85.82	95.62	100	633.4	4	Epitope 1	11=
3/11	97.57	72.57	86.88	94.94	85.82	95.62	100	633.4	4	Epitope 1	11=
Mab24	91.08	63.21	84.21	85.73	86.67	80.96	100	591.86	2	Epitope 1	15
HC84.22	59.59	4.2	0	0.92	0	0.51	0	65.22	0	Epitope 1, 2, and 4	32=
HC84.23	59.59	4.2	0	0.92	0	0.51	0	65.22	0	Epitope 1, 2, and 4	32=
AR3C	42.23	28.13	15.14	46.2	7.69	13.59	0	152.98	0	Epitope 2 and 3	28
e20	59.59	4.2	0	0.92	0	0.51	0	65.22	0	Epitope 2, 3, and 4	32=
HC-11	42.23	4.2	0	0.92	0	0.51	0	47.86	0	Epitope 2 and 3	36=
HC-1	99.78	98.49	86.15	99.15	100	98.62	100	682.19	5	Epitope 3	7
HC84.20	99.81	99.58	99.8	99.51	100	100	100	698.7	4	Epitope 2 and 4	1
HC84.21	81.85	99.12	74.83	96.08	20.51	90.47	100	562.86	4	Epitope 2	16=
HC84.24	81.85	99.12	74.83	96.08	20.51	90.47	100	562.86	4	Epitope 2	16=
HC84.25	81.85	99.12	74.83	96.08	20.51	90.47	100	562.86	4	Epitope 2 and 4	16=
HC84.27	69.80	16.37	74.83	85.60	20.51	66.97	0	334.08	0	Epitope 2 and 4	24
mAb#8	42.23	4.2	0	0.92	0	0.51	0	47.86	0	Epitope 2	36=
mAb#41	42.23	4.2	0	0.92	0	0.51	0	47.86	0	Epitope 2	36=
CBH-2	59.59	4.2	0	0.92	0	0.51	0	65.22	0	Epitope 2 and 3	32=
HC84.1	81.85	99.12	74.83	96.08	20.51	90.47	100	562.86	4	Epitope 2	16=
HC84.26	81.85	99.12	74.83	96.08	20.51	90.47	100	562.86	4	Epitope 2	16=
1:7	95.81	98.49	86.15	97.86	94.74	98.62	100	671.67	6	Epitope 3	9=
A8	95.81	98.49	86.15	97.86	94.74	98.62	100	671.67	6	Epitope 3	9=
Mab44	98.73	99.43	4.03	99.51	100	0.56	100	502.26	5	Epitope 3 and 4	23
J6.27	61.23	25.14	38.41	1.26	0	4.52	0	130.56	0	Epitope 3	30
H77.31	49.09	3.02	2.12	9.83	10.53	1.4	0	75.99	0	Epitope 3	31

*Shading denotes >90% conservation. Each antibody has been ranked from the most to the least conserved according to the sum of the lowest level of conservation (%) across all genotypes. An = symbol shows broadly neutralizing antibodies that have equivalent rank*.

The epitopes of antibodies AR4A and AR5A, which bind residues in E1 and toward the C-terminus of E2, were highly conserved. Epitope 1 is also well-conserved, indeed, the majority of bNAbs that target this region are ranked highly overall. The differences in rank, of the Epitope 1-binding bNAbs, can be attributed to the specific residues recognized by each antibody. For instance, bNAbs AP33, HCV1, H77.39, and 3/11 all bind N415, which has a lower level of conservation in gt2, gt3, and gt5. This reduces the level of conservation of these antibodies compared to HC33.1, 95-2 or Hu5B3.v3, which do not require N415 for binding. Similarly, the Mab24 epitope is not as well conserved due to its requirement for T416. For the HC33 group of antibodies, those that require K408 are ranked lower overall as this residue, which is located in the HVR1, perhaps unsurprisingly, is poorly conserved. However, in contrast, the residues 403 and 406 in HVR1 that are recognized by J6.36 and J6.103 are unexpectedly well-conserved (>90% conservation in gt4, gt5, and gt6).

The bNAbs that bind to Epitope 2 generally perform less well, the exception is HC84.20, which ranked at the top of Table 2. Many of the HC84 series of the bNAbs are the highest ranking Epitope 2-interacting antibodies. There is >90% conservation of the binding motif within this region of E2 of gt2, gt4, and gt6, whereas the corresponding residues in the gt1 and gt3 glycoproteins are less conserved (81.85 and 74.83%, respectively). This is even more striking in gt5 where conservation of the binding motif is only 20.51%. These reductions are attributable to the phenylalanine at position 442 of the binding motif; this hydrophobic residue is changed to an aliphatic residue in a significant proportion of sequences. The particular aliphatic residue varies depending on genotype, e.g., leucine (12.38%) in gt1, isoleucine (18.6%) in gt3, and methionine (35.9%) in gt5. This change is likely to affect antibody binding. HC84.20 ranks so highly because F442 is not a critical binding residue for this bNAb. The HC84.22 and HC84.23 binding motifs are poorly conserved, due to their interaction with W437. This residue also forms part of the binding motif for other bNAbs that interact with Epitope 2, CBH-2, mAb#8, mAb#41, and e20. However, in the majority of instances (>95% for all but gt1), it is replaced by the hydrophobic phenylalanine residue: this amino acid has very similar properties; therefore, it is likely that a degree of antibody binding would be retained. Remarkably, the binding motif of the strongly neutralizing bNAb AR3C has a low level of conservation across all genotypes. Further inspection of the sequences shows that this is due to L438, which is replaced by an isoleucine in the majority of sequences. This is a conservative change; therefore, it is probable that antibody interaction may be retained.

### Investigation of bNAb Epitopes in HCV Subtypes

The bNAbs with the most conserved binding motifs were further analyzed for the level of conservation at the HCV subtype level. For the analysis, we selected the 10 best represented subtypes in the dataset (1a, 1b, 2a, 2b, 2c, 3a, 3b, 4a, 4d, and 6a). This scrutiny revealed some interesting observations at the subtype level, as shown in Table 3. Surprisingly, the conservation of the binding motif of bNAbs HC-1, 1:7 and A8 was 0% in the 3b subtype compared to 99.09% for gt3a indicating that these antibodies may not neutralize gt3b. Further examination of the epitope sequence showed that this is due to replacement of W529 to a phenylalanine in all gt3b sequences (*n* = 351) in the database. This is a relatively conservative change from one hydrophobic residue to another; therefore, it is probable that the bNAbs will still bind gt3b. Likewise, further dissection of the genotypes into subtypes for the Epitope 1-binding bNAbs AP33, HCV1, H77.39 and 3/11 highlighted differences at the subtype level for both gt2 and gt3. This is due to the asparagine residue at position 415. While in gt2a, there is a high level of conservation at this position (97.27%), this drops to 82.94% in gt2b and more substantially to only 39.47% in gt2c. While in gt2b, this drop is due to an increase in the presence of serine at this position, the majority of gt2c sequences (57.68%) were found to have a histidine residue instead. Serine and asparagine are both hydrophilic, neutral residues and therefore this change may not substantially affect antibody binding. Histidine, however, is positively charged and this more dramatic change, found in gt2c, is more likely to abrogate antibody interaction. Similarly, in gt3a, there is a drop in conservation of N415 to 86.88%. This decrease is due to a higher prevalence of the positively charged arginine residue at this position, which would also be predicted to inhibit antibody binding.

**Table 3:** The lowest level of conservation (%) for each bNAb epitope-binding motif across subtypes.

Name	Gt1a% Con	Gt1b% Con	Gt2a% Con	Gt2b% Con	Gt2c% Con	Gt3a% Con	Gt3b% Con	Gt4a% Con	Gt4d% Con	Gt6a% Con	Region targeted
HC84.20	98.58	99.73	98.48	99.72	100	99.79	99.75	99.24	100	100	Epitope 2 and 4
AR4A	99.76	99.57	99.8	99.7	100	99.74	98.13	98.13	99.23	100	E1E2
AR5A	99.76	99.57	99.8	99.7	100	99.76	100	98.13	99.23	100	E1E2
95-2	99.66	99.83	100	98.7	100	99.91	99.75	100	100	99.7	Epitope 1
HC33.1	99.55	99.83	100	98.7	100	99.82	99.75	100	99.82	99.7	Epitope 1
HC33.32	99.55	99.83	100	98.7	100	99.82	99.75	100	99.82	99.7	Epitope 1
HC-1	99.78	99.66	92.21	99.72	98.44	99.09	0	99.38	100	100	Epitope 2 and 3
1:7	94.62	98.87	92.21	99.43	98.44	99.09	0	97.5	100	100	Epitope 3
A8	94.62	98.87	92.21	99.43	98.44	99.09	0	97.5	100	100	Epitope 3
Hu5B3.v3	94.46	98.44	93.49	98.7	96.93	96.86	99.51	98.77	99.45	98.81	Epitope 1
AP33	96.98	98.49	97.27	82.94	39.47	84.85	97.8	100	93.91	95.87	Epitope 1
HCV1	96.98	98.49	97.27	82.94	39.47	84.85	97.8	100	93.91	95.87	Epitope 1
H77.39	96.98	98.49	97.27	82.94	39.47	84.85	97.8	100	93.91	95.87	Epitope 1
3/11	96.98	98.49	97.27	82.94	39.47	84.85	97.8	100	93.91	95.87	Epitope 1

*Shading denotes > 90% conservation*.

### Analysis of the Binding Motif Pattern

The conservation analysis reported above treats each position within the antibody motif as a single entity. This approach would not identify changes within the binding motif pattern that modify more than one substitution within the epitope. To investigate this, we analyzed the complete binding motif pattern for the 10 bNAbs with the most conserved epitopes (Table 4; Table S2 in Supplementary Material). The most prevalent amino-acid patterns, i.e., found in at least 10 sequences are shown in Table 4. These data confirm the high level of conservation of these epitopes across all seven genotypes. In all but one example, the most prevalent sequences only had a single substitution within the binding motif. For bNAbs AR4A and AR5A, the level of epitope conservation is particularly dramatic. From over 9,700 sequences analyzed, there was only a single alternative sequence that was present in at least 10 sequences. This had a single conservative change at E1 204 replacing threonine with serine. The HC84.20 epitope motif was also very highly conserved with a single alternative sequence present in 1.1% of gt1 sequences.

**Table 4:** The most prevalent sequences for the complete bNAb epitope motifs.

bNAb	Sequence	Percentage of sequences (%)							Total no. sequences	Gt range
		Gt1	Gt2	Gt3	Gt4	Gt5	Gt6	Gt7		
HC84.20	C/L/Y/W	98.6	99.2	99.5	99.5	100	100	100	11,379	1–7
	C/L/H/W	1.10	0	0	0	0	0	0	89	1

AR4A	Y/TND/R/L/D	99.3	99.2	99.2	97.5	100	99.6	100	9,704	1–7
	Y/SND/R/L/D	0.11	0	0	1.97	0	0	0	12	1, 4

AR5A	Y/TND/R/R	99.5	99.5	99.4	97.5	100	99.1	100	9,741	1–7
	Y/SND/R/R	0.11	0	0	1.97	0	0	0	12	1, 4

95/2	L/W	99.6	99.6	99.9	100	96.3	99.7	100	29,658	1–7
	L/R	0.15	0.07	0.05	0	0	0.17	0	39	1, 2, 3, 6
	P/W	0.07	0	0.05	0	0	0	0	19	1, 3
	L/^a^	0.05	0.07	0	0	0	0	0	12	1, 2
	L/C	0.04	0	0	0	0.75	0	0	11	1, 5

HC33.1 and HC33.32	L/G/W	99.3	99.3	99.7	99.9	94.8	99.5	100	29,565	1–7
	L/N/W	0.19	0	0.03	0	0	0	0	45	1,3
	L/G/R	0.15	0.07	0.05	0	0	0.17	0	39	1, 2, 3, 6
	L/D/W	0.07	0	0.10	0	0	0	0	21	1, 3
	P/G/W	0.07	0	0.05	0	0	0	0	19	1, 3
	L/S/W	0.05	0.07	0	0	0.75	0.17	0	15	1, 2, 5, 6
	L/G/^a^	0.05	0.07	0	0	0	0	0	12	1, 2
	L/G/C	0.04	0	0	0	0.75	0	0	11	1, 5

HC-1	C/WG/D	99.4	98.1	85.7	98.3	100	98.6	100	13,764	1–7
	C/FG/D	0.04	0	13.4	0.43	0	1.08	0	380	1, 3, 4, 6
	C/WG/G	0.12	0	0	0	0	0	0	12	1
	C/RG/D	0.07	0	0.11	0	0	0	0	10	1, 3

1:7 and A8	G/TY/WG/D	91.9	97.5	82.9	96.6	94.7	97.2	100	12,806	1–7
	G/TF/WG/D	3.64	0	2.6	2.14	0	1.10	0	453	1, 3, 4, 6
	G/TY/FG/D	0.02	0	13.4	0.43	0	1.10	0	377	1, 3, 4, 6
	G/AY/WG/D	3.33	0.19	0	0	0	0	0	341	1, 2
	G/TH/WG/D	0.37	0.19	0.07	0	0	0	0	41	1, 2, 3
	G/TY/WG/G	0.11	0	0	0	0	0	0	11	1

Hu5B3.v3	L/N/W/I	95.0	96.1	96.1	97.7	92.5	87.0	100	27,806	1–7
	L/N/W/L	2.37	0.07	0.03	0.65	0	0.51	0	555	1, 2, 3, 4, 6
	L/N/W/V	1.40	2.59	2.70	1.30	0	11.3	0	537	1, 2, 3, 4, 6
	L/S/W/I	0.40	0.37	0.23	0	0.75	0.34	0	109	1, 2, 3, 5, 6
	L/D/W/I	0.16	0.15	0.05	0	0	0.17	0	42	1, 2, 3, 6
	L/N/R/I	0.13	0.07	0.05	0	0	0.17	0	35	1, 2, 3, 6
	L/Q/W/I	0	0	0.62	0	1.50	0	0	26	3, 5
	L/N/W/T	0.10	0.07	0.05	0	0	0	0	25	1, 2, 3
	P/N/W/I	0.07	0	0.05	0	0	0	0	15	1, 3
	L/R/W/I	0.05	0	0	0	0	0	0	12	1
	L/N/^a^/I	0.05	0.07	0	0	0	0	0	12	1, 2
	L/N/C/I	0.04	0	0	0	0.75	0	0	11	1, 5

*^a^Signifies a stop codon, sequence differences are shown in bold*.

The bNAb-binding motifs in Epitope 1 bound by 95/2 and HC33.1 and HC33.32 were marginally more variable; however, each alternative sequence was still represented at a low frequency (fewer than 50 copies) in the database. There was more sequence variation across the Hu5B3.v3-binding motif in Epitope 1, with 12 different sequences identified. For all the Epitope 1-interacting bNAbs, there was no obvious preference for mutations at specific positions as substitutions were found to occur at all residues in the bNAb epitopes and all but one sequence was reported in at least two genotypes. Similarly, in Epitope 3 where the HC-1, 1:7, and A8 binding motifs share three common residues (W529, G530 and D535), there was no pattern for which residue in the binding motif was altered. Gt3 sequences had the lowest frequency of conservation, due to the substitution of W529 with a phenylalanine residue.

### Geographical Distribution

**Figure 5:**
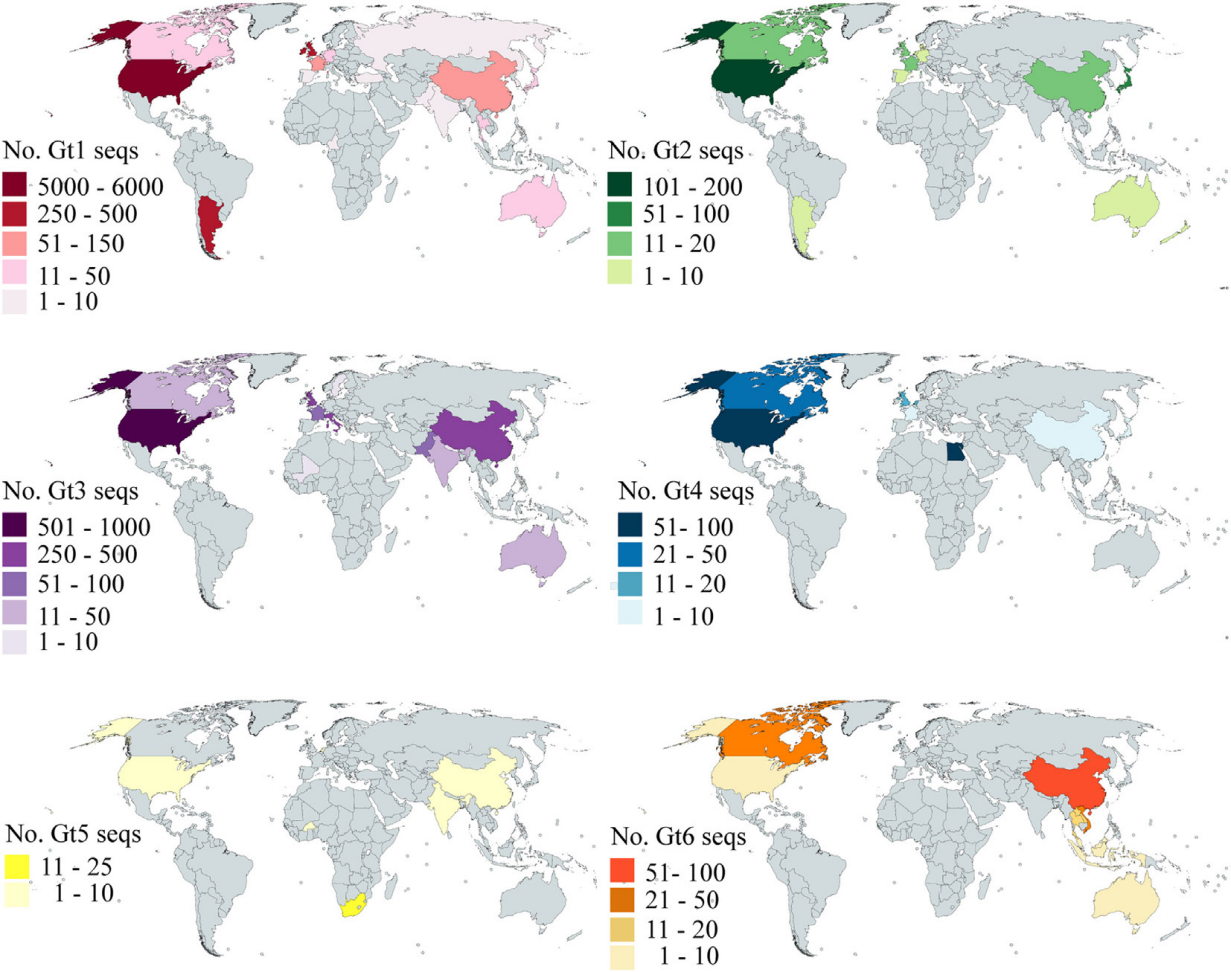
Global distribution of E2 sequence coverage. The maps are color-coded to show the number of E2 sequences (with at least 90% coverage) per country for Gt1 (red), Gt2 (green), Gt3 (purple), Gt4 (blue), Gt5 (yellow), and Gt6 (orange). Countries shaded in gray have no sequences reported.

Our analysis is based on the sequence data available in Genbank. To investigate how the available data reflected the global distribution of HCV, we analyzed the country of origin of the deposited sequences. We focused on E2 as this is the principal target of bNAbs; however, the data for both E1 and E2 are shown in Table S3 in Supplementary Material. The number of sequences that cover at least 90% of E2 for gt1–6 per country is shown in Figure 5. Genotype 7 is not shown as there are only two sequences, both of which were isolated in Canada. There are an additional 1,320 sequences representing gt1–6 where no country is specified. Even for gt1, the most prevalent genotype, while the USA is well-represented, large areas of the globe have severely limited or no sequence information for this region of the HCV genome. Indeed, there are no sequences for Central America, the Middle East, most of the African continent, Eastern Europe, and South America. This trend is even more pronounced with regard to the less prevalent genotypes. There are approximately 3,000 more E1 sequences available compared to E2; however, the overall trend with respect to the global distribution is similar (Figure S1 in Supplementary Material).

## Discussion

The development of the GLUE software has enabled rapid analysis of large datasets of viral sequences (28). HCV-GLUE is the most advanced project; however, similar data resources for other viruses including HIV and HBV are under development (R. Gifford and J. Singer, personal communication). In this study, we have assessed the level of conservation of the critical residues of HCV glycoproteins that are recognized by a panel of HCV bNAbs using HCV-GLUE. This analysis has identified a group of bNAbs that theoretically, based on epitope conservation, would be the best leads for a vaccine design strategy. However the presence of the epitope sequence is not the only factor dictating the efficacy of an antibody; the affinity of the antibody for its target is also important. We and others have noted that some isolates of E1E2 are more resistant to neutralization either by patient sera or purified bNAbs (12, 55–58). Indeed, two of these studies, which both performed large-scale neutralization studies of >80 E1E2 isolates identified three groups, with those that were either highly sensitive or highly resistant at the extremes and the majority falling somewhere in between. The reason for this is unclear, Urbanowicz and coworkers suggest that multiple mechanisms are likely to be involved as they could not identify any common sequence substitutions that explained the extreme phenotypes (55). In contrast, El-Diwany and coworkers demonstrated that two polymorphisms at positions 403 and 438 modulate neutralization phenotype and suggest that this is *via* alteration of binding to the receptor SR-BI (57). Moreover, in the context of an HCV infection, it has been demonstrated that the glycan shield formed by extensive glycosylations of E1E2 can prevent bNAbs binding to their neutralizing epitopes (14, 59). Studies also indicate that the HVR1 of E2 functions to block access to neutralizing epitopes, this is variable between E1E2 isolates strongly influencing the neutralization phenotype (12). Also relevant in the case of HCV, which is particularly diverse in sequence, is the possibility that a contributing factor to the breadth of binding by bNAbs may be due to the antibody paratope being able to accommodate alternative residues. Therefore, we examined the available neutralization data for the bNAbs with the most conserved epitopes.

By definition, all bNAbs can neutralize more than one viral genotype, although they have been assessed by different groups in different systems with different isolates and methods and thus it is difficult to compare the results directly. With regards to the conservation of the epitope sequence, we identify HC84.20 as the top candidate; it has been assessed for neutralization activity in the HCV cell culture (HCVcc) system and was able to neutralize all E1E2 sequences (gt1–6) except for gt3. AR4A, which ranked second overall performed better in neutralization studies reported by Giang et al. (32). AR4A was able to neutralize the full complement of E1E2 sequences (gt1–6) tested in both the HCVpp and HCVcc systems. The AR4A epitope was conserved in all 24 sequences tested. AR4A was able to neutralize 85.8% of E1E2 isolate in a large-scale neutralization study (57). According to our data, as the AR5A epitope is also conserved in all the sequences tested, it should behave similarly. In practice; however, this bNAb could neutralize gt 2 sequences in the HCVcc system but not in the HCVpp system and could not neutralize any gt3 sequence tested (32). From the alanine-scanning mutagenesis, AR4A and AR5A have overlapping epitopes; however, our results together with the neutralization data suggest that AR5A binds additional residues that have yet to be identified (32). This is supported by the observation in the original paper that the epitope of AR5A but not AR4A overlaps with the epitope of the bNAb CBH-7 (32). CBH-7 was not included in our analysis as, despite much effort, the epitope has not yet been defined (27, 60, 61).

Several of the top-ranking bNAbs bind to Epitope 1; of these, bNAb 95-2 has the most conserved epitope composed of only two residues L413 and W420. This antibody was isolated by Broering and coworkers along with bNAb HCV1 (39). They report that both antibodies were able to neutralize all six HCVpp tested (gt 1–4) suggesting broad neutralization, although HCV1 was marginally less effective. There are no data available for gt 5 or 6. The fact that only two residues (L413 and W420) are required by bNAb 95-2 for binding contributes to the high level of conservation. It should be noted that, in the original study, bNAb HCV1 was also reported to only require the same two residues. Subsequent work by Kong et al. (40) who showed by alanine-scanning mutagenesis, and more importantly, by co-crystallization of HCV1 in complex with Epitope 1 peptide that N415 and G418 were also required (40). To our knowledge, no further work with 95-2 has been reported in the literature and therefore it remains possible that 95-2 may also require additional residues for binding. Other Epitope 1 bNAbs HC33.1, HC33.32, and H77.39 do not neutralize as well as we would predict from our results even though their epitopes are conserved in all the sequences tested (33, 34). Contrary to our predictions, in practice, H77.39 is the most effective, neutralizing gt1, gt2, gt4, and gt5 but not gt3 or gt6. HC33.1 did not efficiently neutralize gt2, gt3, or gt6 and HC33.32 performed marginally less well neutralizing only gt2, gt4, and gt5 (33, 34). These data suggest that other factors are influencing antibody performance. As mentioned above, these may be attributes of the antibody themselves such as affinity and avidity. Alternatively, properties of the E1E2 glycoproteins such as glycosylation or HVR1 structure may be preventing access to the epitope. Hu5B3.v3 could neutralize both gt1 and gt2, but it has not been tested against other genotypes (46).

AP33 and 3/11 that both recognize E2 Epitope 1 residues (L413, N415, G418, W420 for AP33 and N415, W420, H421 for 3/11) have been tested against a wider range of E1E2 sequences although these studies were performed prior to the development of the HCVcc system (18, 41). Tarr et al. compared the performance of both antibodies in parallel (41). AP33 performed significantly better than 3/11, neutralizing 17/18 HCVpp (gt 1–6) compared to 6/18 isolates (gt1, gt2, gt4, gt5, and gt6). This is despite the 3/11 epitope being completely conserved, in contrast to that of AP33. Indeed, the sequence that AP33 did not neutralize contained two mutations in the AP33 epitope N415Q and G418S, which explains the lack of neutralization. Notably, a sequence that was neutralized by AP33 had a change in one of the antibody-interacting residues whereby N415 was replaced by a histidine. This is particularly interesting as nearly 60% of gt2c sequences have a histidine at this position. Therefore, this data suggests that AP33 can still bind and neutralize sequences containing this variant, which would increase the predicted level of AP33 binding to gt2 sequences. It has been shown that a mutation N417S/T results in a glycan shift within Epitope 1 from 417 to 415 that can block binding with neutralizing antibodies, such as AP33, which require N415 (37, 62). The shift is due to a change in position 417 to either serine or threonine that creates an N-linked glycosylation site. Consequently, we checked the frequency of these mutations in the database. We find that these mutations have been detected in the majority of genotypes, with the exception of gt4 and gt7, albeit at low frequency (0.23–0.74%). AP33 performed well in a large-scale neutralization study with a mean IC_50_ value of 0.69 µg/ml (55). Hu5B3.v3 has been shown to neutralize gt1 and gt2 sequences, but there is no data for other genotypes (37).

The Epitope 3-binding antibodies 1:7 and A8 both have been shown to perform well in neutralization studies. They were tested against 10 different E1E2 sequences (gt1–6) in the HCVpp system (54). The epitope was conserved in all the sequences. 1:7 was marginally better than A8 neutralizing HCVpp bearing all 10 sequences by at least 50%. A8 only neutralized 9/10 isolates by >50%; however, this was at a relatively low concentration (15 µg/ml) compared to other studies. 1:7 was included in a large-scale neutralization study and shown to have a mean IC_50_ value of 2.1 µg/ml (55). HC-1, which binds in part to Epitope 3 did not function as well in neutralization tests. Although its epitope is conserved, HC-1 only neutralized 3/8 E1E2 sequences (gt1 and gt 5) tested (63). Similarly to bNabs 1:7 and A8, the highest concentration tested was 20 µg/ml compared to 50 µg/ml for other studies; therefore, this might improve at higher concentrations. From our bioinformatics analysis, the top bNAbs would be HC84.20, AR4A, AR5A, and 95/2; however, if we take into account the available neutralization data we can conclude that HC84.20, AR4A, 1:7, A8, and AP33 are the most promising lead candidates to date. 95-2, HCV1, and Hu5B3.v3 performed well in our analysis but have not been tested as extensively across different genotypes for neutralization. AR5A, HC33.1, HC33.32, HC-1, H77.39, and 3/11 all have highly conserved epitopes; however, they were not as effective in neutralization studies.

One unexpected result from our analysis was the relatively low level of conservation of the AR3C antibody epitope residues, this was principally due to the requirement for L438. This residue is poorly conserved with isoleucine and valine being the main variants. Our data are in stark contrast to the neutralization studies reported for AR3C (24, 64). AR3C neutralized 27/29 E1E2 sequences tested in the HCVpp system (24, 64). Examination of the sequences showed that several isolates had either isoleucine or valine at position 438, thereby providing strong evidence that AR3C can still bind and neutralize these variants. If we adjust for this in our analysis, the level of conservation is significantly improved, indeed, AR3C would be ranked 16th overall.

The analysis of the bNAb epitope motifs shows that there is a strong preference for the bNAb epitope consensus sequence, there are very few variants circulating at significant levels. This makes it feasible to assess whether the major variants remain susceptible to neutralization. Indeed, only 30 variants would be required to test this for the most conserved bNAbs as the epitopes have several residues in common.

A recent publication by Messina et al. was the first to estimate the relative prevalence of HCV globally (65). Prevalence was estimated in 20 geographical regions and by country, data permitting. Gt1 was the most prevalent in 15/20 geographical regions accounting for 46.2% of cases. Nearly one-third of cases worldwide are due to gt3. The remaining ~25% are due to gt2, gt4, and gt6. Of these, gt2 is the most widespread globally although it does not have the highest prevalence in any region. Gt4 is localized mainly to Africa, indeed, it has the highest frequency in North Africa and the Middle East and Central sub-Saharan Africa. Like gt2, gt6 is not the most prevalent genotype in any region; however, it is significant in East and Southeast Asia. Finally, gt5 accounts for the lowest frequency globally, but is highly localized in southern sub-Saharan Africa. Our data show that the top candidate bNAbs identified here should be effective against the majority of variants. Indeed, the HC84.20 and AR4A epitopes are conserved in >99% of available sequences for each genotype; therefore, a vaccine based on these epitopes would be predicted to be effective against all genotypes. While well-conserved against the most prevalent genotype, gt1, both 1:7 and AP33 epitopes are less well conserved in gt3. However, in the case of 1:7, this was specifically gt3b sequences whereas AP33 epitope residues were more conserved in gt3b than gt3a suggesting that these antibodies may complement each other. In the same way, conservation of the AP33 epitope was reduced in gt2b and gt2c; however, the 1:7 epitope was highly conserved in these subtypes.

## Limitations

There are some caveats to the analysis reported in this study. The first is that our analysis is based on the publicly available sequence data available in Genbank. HCV sequence data in GenBank captures the known genomic diversity of this virus, and this dataset has for example underpinned the genotype and subtype definitions for the virus (4). HCV sequences may be submitted to GenBank as part of research projects with a wide variety of aims. Large sequence sets for a particular kind of research, for example, focusing on specific patient cohort types, may dominate. GenBank does not, therefore, accurately represent the range of HCV genomic diversity that a vaccine strategy would face “in the wild.” However, GenBank does provide a reasonable number of sequences within certain major genotypes and subtypes and so it does give a view on which genomic patterns are viable for the virus; which can, therefore, be presumed to exist in the wider epidemic. Another factor is that while the developed world has some coverage of the HCV glycoproteins, significant areas of the globe have little or no sequence data available. As a result, particular genotypes such as gt5 and gt6 are poorly represented, and it is possible that other circulating genotypes/subtypes have yet to be identified. To truly understand the extent to which a bNAb-based vaccine may be effective globally, this data gap should be addressed. The analysis also depends on the availability and quality of the bNAb epitope mapping data. Obviously, bNAbs that have not been finely mapped could not be included in our analysis. Although the number of co-crystallization studies has increased in recent years, the majority of epitope mapping is based on alanine-scanning mutagenesis. While this is an important tool, the results may also identify residues that prevent antibody-interaction due to conformational changes. There are also examples whereby alanine-scanning experiments have not been able to conclusively identify the epitope, for instance, bNab e137 where mutation of 15 different residues prevent antibody binding (46, 47, 66). This study is based on sequence conservation of bNAb epitopes, in practice, other factors including antibody affinity, epitope shielding by both the glycan shield, and HVR1 will all influence the efficacy of each antibody. Furthermore, the epitope residues of many of the bNAbs have been determined by alanine-scanning mutagenesis, which does not unequivocally prove direct contact in the absence of structural information of the antigen/antibody complex. Unfortunately, the neutralization data reported in the literature is also variable for the different bNAbs, the assays have not been standardized and few comparative studies are available.

Despite the limitations, from our study, we can conclude that based on epitope conservation and the available neutralization data that the most promising bNAbs for potential vaccine leads are HC84.20, AR4A, 1:7, A8, and AP33. Our results are encouraging as several bNAb epitopes were highly conserved across all genotypes. This finding supports the notion that a single HCV vaccine could indeed fit all, and that ultimately tailoring vaccines to specific regions may prove to be unnecessary. However, further analysis of how the bNAbs perform in large-scale neutralization trials will be required to conclusively test this. Our analysis of the most prevalent epitope variants in circulation should provide useful information when designing these experiments. Nonetheless to counter escape mutations and provide sterilizing immunity, it is likely to be desirable to develop a multi-target vaccine, this could be a B-cell and T-cell-based combination or a combination of different B-cell targets.

## Author Contributions

Conceptualization (VC and AP), design and development of HCV-GLUE (RG and JS), generation of data (JS), analysis of data (VC), writing—original draft (VC and JS), writing—review and editing (AP and RG), funding (AP and RG).

## Conflict of Interest Statement

The authors declare that the research was conducted in the absence of any commercial or financial relationships that could be construed as a potential conflict of interest. The handling Editor declared a past co-authorship with one of the authors AP.
