# Supplementary figures and images for: Predicting the Effectiveness of Hepatitis C Virus Neutralizing Antibodies by Bioinformatic Analysis of Conserved Epitope Residues Using Public Sequence Data
Source: Front Immunol. 2018 Jun 27;9:1470. doi: 10.3389/fimmu.2018.01470 (PMC6036255; doi:10.3389/fimmu.2018.01470)

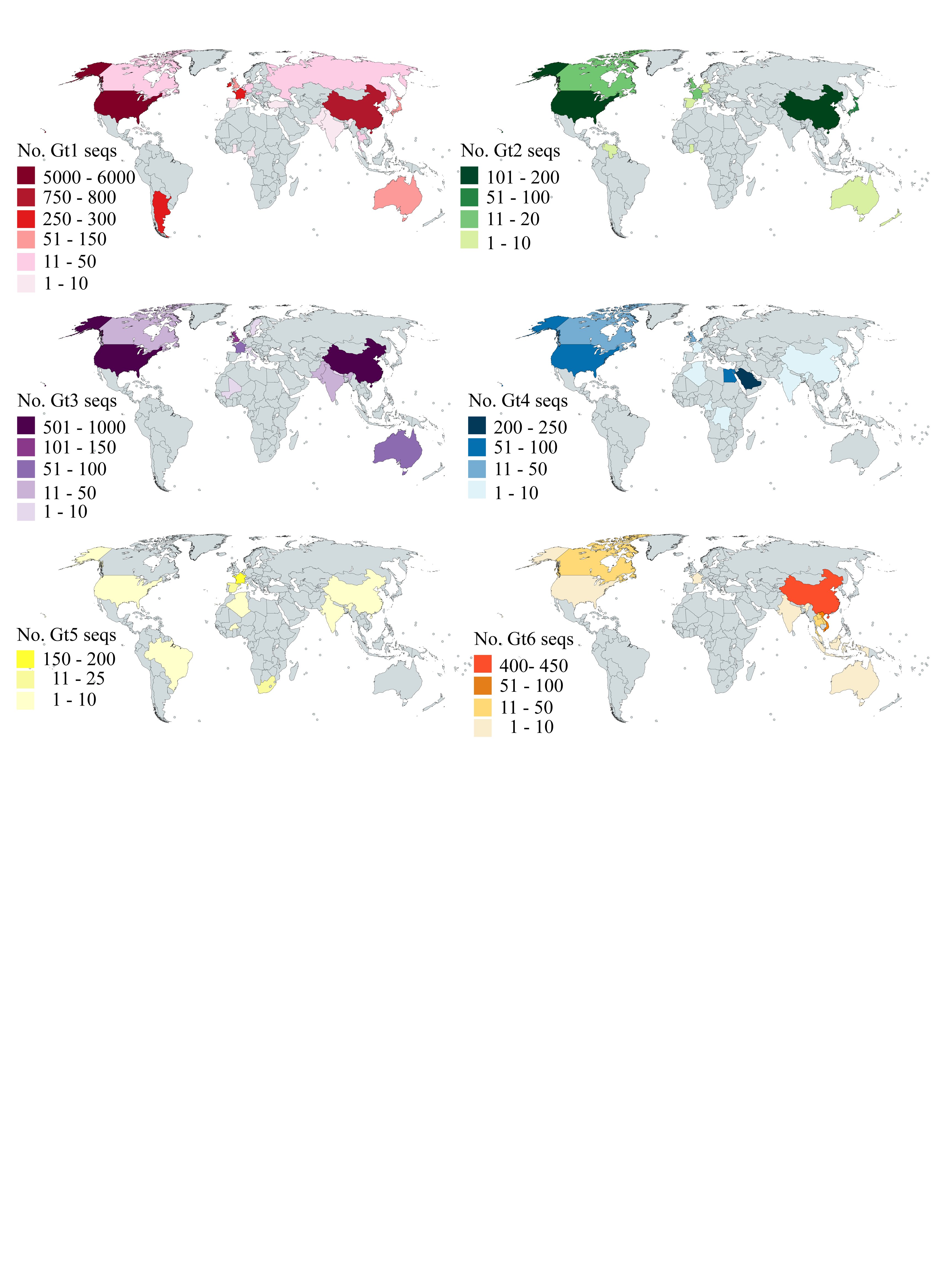

Supplement: Supplementary file 1 [file Image_1.tiff]
